# Comprehensive Analysis of Adverse Events Induced by PARP Inhibitors Using JADER and Time to Onset

**DOI:** 10.3390/life12091355

**Published:** 2022-08-31

**Authors:** Kenta Yamaoka, Masaki Fujiwara, Mayako Uchida, Yoshihiro Uesawa, Nobuyuki Muroi, Tadashi Shimizu

**Affiliations:** 1 Department of Pharmacy, Kobe City Medical Center General Hospital, Kobe 650-0047, Japan; 2 School of Pharmacy, Hyogo Medical University, Kobe 650-8530, Japan; 3 Department of Education and Research Center for Pharmacy Practice, Faculty of Pharmaceutical Sciences, Doshisha Women’s College of Liberal Arts, Kyotanabe 610-0395, Japan; 4 Department of Medical Molecular Informatics, Meiji Pharmaceutical University, Tokyo 204-8588, Japan

**Keywords:** Poly (ADP-ribose) polymerase (PARP) inhibitor, olaparib, niraparib, spontaneous reporting system, Japanese Adverse Drug Event Reporting (JADER), pharmacovigilance

## Abstract

Poly (ADP-ribose) polymerase (PARP) inhibitors are effective against breast cancer susceptibility gene (*BRCA*) mutations. Clinical trials have reported hematologic toxicity and gastrointestinal symptoms as class effects of PARP inhibitors. However, information on adverse events (AEs) in a Japanese clinical cohort is currently lacking. In this study, we conducted a comprehensive survey of the AEs of two PARP inhibitors, olaparib and niraparib, using the Japanese Adverse Reaction Reporting (JADER) database provided by the Pharmaceuticals and Medical Devices Agency (PMDA). Moreover, we also analyzed the course and time to the onset of AEs. Signals were detected for 15 and 11 AEs for olaparib and niraparib, respectively. Most occurred within the first month of treatment with either agent. These results may indicate the importance of early response and monitoring after beginning PARP inhibitor therapy. The results of this study may be useful for managing side effects and suggesting supportive care for patients using PARP inhibitors in the future.

## 1. Introduction

Mortality due to ovarian cancer has increased in Japan in recent years, with an estimated 4876 deaths in 2020, making it the most common fatal malignant tumor of the female genital tract [1]. Pathological variants of the breast cancer susceptibility gene (*BRCA*) have been found in 14.1% of ovarian cancer cases overseas and 14.7% of those in Japan [2,3,4,5]. *BRCA1* and *BRCA2* are genes belonging to the homologous recombination pathway that are involved in DNA damage repair and cell division. Inherited pathological variants of these genes are known to cause a cancer susceptibility syndrome called hereditary breast and ovarian cancer syndrome [6]. In addition to *BRCA1/2*, other genes related to the homologous recombination pathway are known to mutate and cause loss of function, resulting in homologous recombination deficiency [7,8,9,10,11].

Poly (ADP-ribose) polymerase (PARP) inhibitors have been reported to be effective against hereditary breast and ovarian cancer syndrome and homologous recombination deficiency, and olaparib was first approved in Japan in July 2018, followed by niraparib [12,13]. PARP inhibitors are currently approved for the treatment of ovarian and breast cancer, as well as for prostate and pancreatic cancer, albeit under certain conditions [14,15,16,17,18,19,20,21]. PRIMA and NOVA studies of the efficacy and safety of niraparib showed similar incidence and severity of side effects, with good adherence to treatment [22,23,24]. Reported class effects of PARP inhibitors include myelosuppression such as thrombocytopenia, anemia, and neutropenia; gastrointestinal symptoms such as nausea and vomiting; and renal dysfunction [25]. While adverse events (AEs) were common, most were mild to moderate AEs in clinical trials, and treatment could be continued by using prophylaxis or reducing the dosage [14,15,18]. In addition, analysis using the FDA Adverse Event Reporting System (FAERS) revealed a similar trend to clinical trials, with AEs such as acute myeloid leukemia (AML) and myelodysplastic syndrome (MDS) for olaparib and blood pressure changes and photosensitivity being most common for niraparib [15,19,26]. Studies on Asian patients have generally shown similar results, with most patients experiencing AEs. Treatment could still be continued with alterations, including dose reduction [27].

However, to date, there has been no comprehensive analysis of AEs associated with PARP inhibitors in Japanese clinical practice. In addition, the number of days to onset and subsequent outcomes of AEs have not been clarified. Since the indication of PARP inhibitors has been expanded to several cancer types and the number of patients using PARP inhibitors is expected to increase in the future, a comprehensive analysis of AEs would provide significant insights into the routine application of these drugs in the future. Using a spontaneous adverse event reporting database to identify safety signals may be a valid way to generate hypotheses about the possible relationship between unknown or latent AEs and medications. Here, we analyzed AEs that showed adverse event signals during the administration of olaparib or niraparib using the Japan Adverse Event Reporting (JADER) database published by the Pharmaceuticals and Medical Devices Agency (PMDA). Further, we clarified the number of days until the occurrence of these AEs and their outcomes.

## 2. Materials and Methods

### 2.1. JADER and Production of the Data Analysis Table

The JADER data are publicly available and can be downloaded from the PMDA website (https://www.pmda.go.jp: accessed on 6 June 2022). The study used data reported from April 2004 to December 2021. The JADER database consists of four tables: patients’ demographic information (DEMO), drug information (DRUG), AE information (REAC), and primary disease information (HIST). The “DEMO” section contains basic patient information such as gender, age, and reporting year; the “DRUG” section contains information on the drug (generic name), trade name, route of administration, start date of administration, end date of administration, and drug involvement. The “REAC” section contains information on AEs, including the name of the AE, its onset date, and outcome, and the “HIST” section contains information on the patient’s underlying disease.

AE names were analyzed based on the preferred terms (PTs) listed in the Japanese version of the International Conference on Harmonisation’s International Glossary of Terms for Medicinal Products, ver. 24.1 (https://www.jmo.pmrj.jp/ accessed on 22 June 2022). The MedDRA dictionary is organized with a five-level hierarchy, including system organ class (SOC), high-level group term (HLGT), high-level term (HLT), PT, and lowest level term (LLT). PTs represent more precise medical terminology.

### 2.2. Association of PARP Inhibitors with Adverse Events

Olaparib and niraparib were included in the analysis, and signal estimation was performed using a 2 × 2 contingency table divided by the presence or absence of the suspect drug and the presence or absence of AEs, with the ROR calculated [28]. A signal was detected when the lower limit of the 95% CI of the calculated ROR was >1. The outcome of AEs for which a signal was detected was analyzed.

### 2.3. Time to Onset Analysis

To identify the AE onset time, the following formula was used, in which data with missing “date of start of administration” and “date of onset” in the database were removed.

Adverse event onset date = “date of onset of adverse event” − “date of start of treatment” + 0.5

For cases with multiple dosing start dates, the first dosing start date prior to the onset date of the AE was substituted into the above equation. In this study, the maximum period before the onset of AEs for analysis was two years (730 d).

The number of days to the onset of AEs was fitted to a Weibull distribution on a graph of cumulative incidence rate, where the failure rate was plotted instead of the survival rate, using survival time analysis with the calculated onset time. The Weibull distribution is represented by scale (α) and shape parameters (β). The shape parameter was used to evaluate the profile of the timing of AE onset among these parameters. When β = 1, the hazard is considered constant over time. When β was <1, the hazard was considered to decrease over time (initial failure type). In contrast, when β was >1, the hazard was considered to increase over time (wear-out failure type) [29]. Statistical analysis was performed using JMP PRO^®^15 (SAS Institute, Cary, NC, USA).

## 3. Results

### 3.1. Signal Detection of Olaparib and Niraparib AEs

The total number of AEs reported in the JADER database from April 2004 to December 2021 was 1,890,222. Of these, 1552 and 549 AEs were reported for olaparib and niraparib, respectively. The reported odds ratio (ROR) and 95% confidence interval (CI) for AEs with ≥10 reported cases are shown in Table 1. Among the AEs analyzed, 15 and 11 AE signals were detected for olaparib and niraparib, respectively. Cancer progression and AEs that suggest a possible inadequate response were observed.

In this study, 1287 (83.0%) reports of AEs due to olaparib use were associated with treatment of ovarian cancer and 177 (11.4%) with treatment of breast cancer. In addition, since niraparib is currently approved only for ovarian cancer treatment in Japan, almost all the reported data were related to its use for ovarian cancer, although some off-label use for breast cancer was also reported.

### 3.2. Outcome Analysis after AEs

Of the AEs for which a signal was detected in the previous analysis, we performed another analysis using outcome data registered after the onset of the AEs.

For olaparib, MDS (26.7%), AML (50.0%), malignant neoplasm progression (13.2%), and death (100%) occurred in more than 10% of reported cases (Table 2). In contrast, >40% of patients with anemia (45.0%), decreased platelet count (53.1%), pancytopenia (40.0%), nausea (41.2%), vomiting (40.0%), fatigue (80.0%), interstitial lung disease (ILD) (41.1%), and malaise (46.7%) recovered after the onset of AEs.

For niraparib, the only condition that worsened in >10% of reported cases was death (20.0%) (Table 3). In contrast, >40% of patients with thrombocytopenia (45.4%), decreased platelet counts (48.0%), and decreased neutrophil counts (48.0%) recovered after the AE.

### 3.3. Time to Onset Analysis and Analysis by Weibull Distribution of Each AE

**Figure 1:**
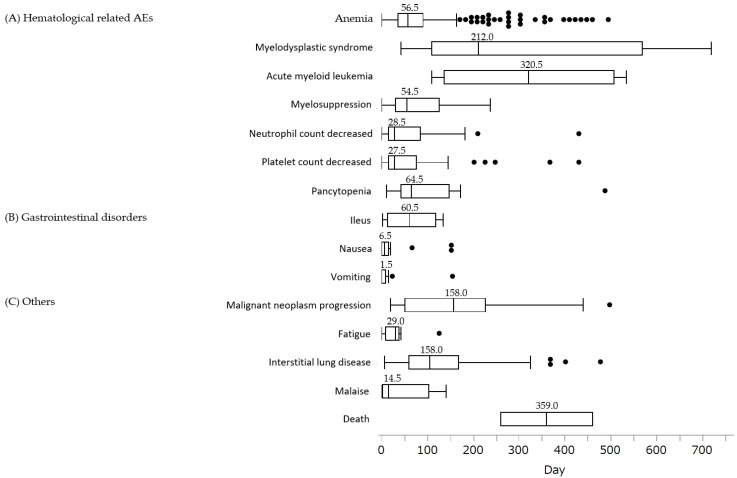
Box chart for time to onset of AEs associated with olaparib. (**A**) Hematological-related AEs: anemia (n = 327), myelodysplastic syndrome (n = 18), acute myeloid leukemia (n = 4), myelosuppression (n = 15), neutrophil count decreased (n = 31), platelet count decreased (n = 41), pancytopenia (n = 14); (**B**) gastrointestinal disorders: ileus (n = 5), nausea (n = 27), vomiting (n = 14); (**C**) others: malignant neoplasm progression ( n = 24), fatigue (n = 8), interstitial lung disease (n = 67), malaise (n = 13), death (n = 2). Box plots show the 25th quartile, 75th quartile, and median (the horizontal line inside the box). The whiskers reach the maximum and minimum values within 1.5 times the inner quartile point’s length. The box’s outside values represent the median number of days before each AE occurred.

The analysis of time from the start of olaparib administration to the onset of AEs is shown in Figure 1. The median (interquartile range, IQR) number of days to AEs for olaparib was 1.5 (0.5–7.5) d for vomiting, 6.5 (0.5–12.5) d for nausea, and 14.5 (1.5–97.5) d for fatigue. Most AEs were concentrated in the first 2 months of treatment. However, AEs that occurred long after treatment initiation were also identified, including interstitial lung disease, 104.5 (74.5–140.5), myelodysplastic syndrome, 212 (112.5–518.5), acute myeloid leukemia, 320.5 (109.5–532.5), and death, 359 (258.5–459.5).

**Figure 2:**
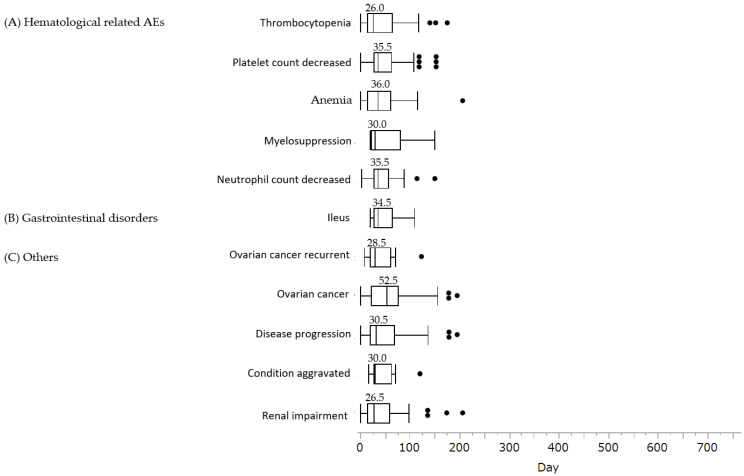
Box chart for time to onset of AEs associated with niraparib. (**A**) Hematological-related AEs: thrombocytopenia (n = 34), platelet count decreased (n = 65), anemia (n = 30), myelosuppression (n = 8), neutrophil count decreased (n = 23); (**B**) gastrointestinal disorders: ileus (n = 10); (**C**) others: ovarian cancer recurrent (n = 20), ovarian cancer (n = 28), disease progression (n = 43), condition aggravated (n = 8), renal impairment (n = 32). Box plots show the 25th quartile, 75th quartile, and median (the horizontal line inside the box). The whiskers reach the maximum and minimum values within 1.5 times the inner quartile point’s length. The box’s outside values represent the median number of days before each AE occurred.

An analysis of the time to onset of niraparib-associated AEs associated with niraparib is shown in Figure 2. All 11 AEs for which a signal was detected tended to occur within two months of initiation of treatment.

The results of the drug-specific analysis by Weibull distribution are shown in Table 4 and Table 5. For olaparib, nausea, 0.54 (0.41–0.070); vomiting, 0.51 (0.34–0.72) were early failure types with an upper limit of 95% CI of <1 for the shape parameter β and a lower failure rate over time. For olaparib, anemia, 1.15 (1.06–1.24); interstitial lung disease, 1.46 (1.21–1.73) were aberrant failure types with a lower limit of 95% CI for shape parameter β of >1, with an increasing failure rate over time.

Niraparib was associated with decreased platelet count, 1.31 (1.07–1.57); ileus, 1.90 (1.12–2.88); recurrent ovarian cancer, 1.50 (1.05–2.01), which were abrasion failure types with the lower limit of 95% CI for the shape parameter β being >1.

## 4. Discussion

### 4.1. Hematologic Toxicities

Hematologic toxicities often lead to either postponement or discontinuation of chemotherapy. Clinical trials have reported that PARP inhibitors require either treatment discontinuation or dose reduction if patients present with hematologic toxicities such as anemia, thrombocytopenia, and neutropenia [14,15,18,19,22,30]. Additionally, clinical trials have shown that thrombocytopenia occurs more frequently with niraparib than with olaparib in cases of relapse, although the frequency of occurrence is similar for both drugs in first-line treatment [14,15,18,19,30]. Hematologic toxicity is also a common AE in clinical trials in Asian patients [27].

In this study, both olaparib and niraparib showed a trend toward more reported hematologic AEs than other AEs. Olaparib showed hematologic toxicity three months after initiation of treatment in the PAOLA-1 study and other studies, whereas this study suggested that it occurred earlier [14,15]. Studies using VigiBase, FAERS, and the World Health Organization pharmacovigilance database suggest that it occurs at around 2 months, and in the present study, we suggested a similar time frame [31,32].

However, hematologic toxicity associated with niraparib developed after one month of the administration, similar to the PRIMA study and others [18,19]. A systematic review and network meta-analysis showed that among PARP inhibitors, niraparib is the most hematologically toxic, and caution is needed in Japanese patients [33]. Other reports have also suggested that thrombocytopenic events occur more frequently with niraparib and soon after the start of treatment in patients with a baseline body weight of less than 77 kg and platelet count of 150,000 cells per mL, and the incidence of grade 3 or higher AEs is lower when the initial dose is reduced to 200 mg once daily, compared with those with olaparib; this indicator should be considered with greater caution in small-bodied Japanese patients compared with that in westerners [23,34,35]. In clinical trials, many patients were able to continue treatment with PARP inhibitors after blood transfusions, use of erythropoietin, and withdrawal or dose reduction; thus, appropriate monitoring is vital. Particular attention should be paid from the beginning of treatment, as hematologic toxicity tends to develop around one month after switching to a PARP inhibitor. 

Although AML or MDS occurrence was not found in the subset analysis of Japanese patients in the PAOLA-1 trial, they were detected as AEs in this study, similar to that in other clinical trials and in the same Asian population [14,15,19,26,27,36,37,38]. However, AML and MDS have been reported to occur less frequently with the use of other PARP inhibitors [30]. Furthermore, the development of AML or MDS has been reported in patients with solid tumors, such as ovarian or breast cancer, either with or without the use of PARP inhibitors [39,40]. Furthermore, the median times from the start of PARP inhibitor administration to the onset of disease were approximately 7 and 11 months for MDS and AML, respectively, which is earlier than in previous reports (17.8 months). This suggests that the disease may develop long after administration [36]. Some reports also indicated the disease occurred after treatment was completed [15,19]. Based on these results, the PARP inhibitor may not be the responsible for AML and MDS. In clinical practice, however, long-term monitoring during and after the use of PARP inhibitors is recommended.

### 4.2. Gastrointestinal Disorders

Here, AE signals of nausea and vomiting were detected. Gastrointestinal symptoms are one of the typical AEs with PARP inhibitors, which are classified as moderate to high emetic risk agents in the NCCN Guidelines 2022 [41,42]. The guidelines recommend the use of 5-HT_3_ antagonists such as granisetron and ondansetron as treatments for moderate to high emetic risk [41]. Nausea may be severe in women, young patients, and patients with a history of alcohol consumption and motion sickness. These factors should also be considered when using PARP inhibitors, which are used more frequently in women with ovarian and breast cancers [41]. Use of antiemetics 30 min before bedtime followed by niraparib just before bedtime has been reported as a way to reduce nausea [43]. Treatment with 5-HT_3_ antagonists can often cause constipation [44]. Contrary to this, a meta-analysis of gastrointestinal AEs in ovarian cancer revealed that the incidence of nausea and diarrhea is significantly increased with the use of PARP inhibitors [45].

In the current study, ileus was detected as an AE signal for both olaparib and niraparib, supporting the findings of another report also suggesting that PARP inhibitors can cause gastrointestinal antecedents and obstruction [46]. Other studies report that olaparib dose reduction or discontinuation was most associated with vomiting and nausea [47]. These findings shed light on the importance of noting all side effects when using PARP inhibitors and the use of patient-appropriate antiemetic agents. Furthermore, when antiemetic agents are used concomitantly, it is important to select an agent with attention to constipation symptoms and regularly monitor defecation patterns.

### 4.3. Other Adverse Events

In addition, the study detected renal dysfunction signals, a known class effect of PARP inhibitors, in niraparib [25]. However, no signals were detected for AEs such as elevated blood pressure and photosensitivity, which have been reported in clinical trials and FAERS studies [26]. Insomnia has also been reported following use of niraparib, but no signal was detected for it as an AE in this study [18,19,48]. The reports may differ from other studies, as niraparib has only been available for use in Japan for a short time; therefore, there may not be a sufficient accumulation of related AE reports.

Interstitial lung disease signals have also been detected for olaparib, with a high mortality rate exceeding 10% after onset. An increase in the incidence of non-infectious pneumonia has been reported with the use of PARP inhibitors [49]. The results of the Weibull distribution analysis also suggest that the disease may occur with continued treatment over a long period, highlighting the importance of regular symptom monitoring. 

## 5. Conclusions

The present study had several limitations. First, serious AEs may have been reported preferentially. Second, we excluded reports with missing or insufficient data, such as the date of initiation of administration. Third, the study did not adequately assess late toxicity because the analysis of the onset date of AEs was limited to 730 d. Fourth, niraparib has only been on the market in Japan for about a year, and the number of reported cases is limited. Fifth, the effects of concomitant medications cannot be completely ruled out.

The JADER database does not include chemotherapy regimens, doses, or patient background. Olaparib is currently approved for use as either a single agent or in combination with bevacizumab, whereas niraparib is approved only as a single agent and is considered largely unaffected by other anticancer drugs. The possibility of using some medications as a form of supportive therapy for underlying diseases or AEs cannot be ignored. The JADER data are acquired from health care providers as information on drugs most likely to be involved in the AEs is also provided by them. Therefore, we concluded that the AEs analyzed in this study were largely influenced by the use of PARP inhibitors.

Here, we used the JADER database to perform an extensive assessment of the negative effects of the PARP inhibitors olaparib and niraparib. Additionally, we examined the progression of AEs as well as their onset time. There were 11 and 15 AEs with detectable signals for niraparib and olaparib, respectively. The majority happened within the first month of using either medication. These findings may highlight the significance of monitoring and early response following the start of PARP inhibitor therapy.

## Figures and Tables

**Table 1:** Numbers of reports and ROR and 95% CI of olaparib and niraparib associated with AEs.

Olaparib					Niraparib				
Variable	Cases (n)	Non-Cases (n)	Rate (%)	ROR (95% CI)	Variable	Cases (n)	Non-Cases (n)	Rate (%)	ROR (95% CI)
Malignant neoplasm progression	242	1310	15.59	73.50 (63.90–84.55)	Ovarian cancer recurrent	26	523	4.74	1917.13 (1182.47–3108.217)
Anemia	496	1056	31.96	40.50 (36.37–45.09)	Ovarian cancer	35	514	6.38	500.61 (348.02–720.11)
MDS	30	1522	1.93	11.71 (8.15–16.84)	Disease progression	57	492	10.38	101.85 (77.17–134.42)
Fatigue	10	1542	0.64	6.46 (3.47–12.06)	Thrombocytopenia	44	505	8.01	13.60 (9.99–18.52)
AML	12	1540	0.77	6.33	Ileus	11	538	2	11.99
				(3.58–11.18)					(6.60–21.80)
Ileus	14	1538	0.9	5.24	Condition aggravated	10	539	1.82	10.11
				(3.09–8.88)					(5.40–18.91)
Myelosuppression	33	1519	2.13	3.83	Platelet count decreased	75	474	13.66	8.90
				(2.71–5.40)					(6.98–11.35)
Nausea	34	1518	2.19	3.47	Renal impairment	41	508	7.47	7.45
				(2.47–4.88)					(5.42–10.24)
Neutrophil count decreased	63	1489	4.06	2.85	Anemia	39	510	7.1	6.46
				(2.21–3.67)					(4.66–8.95)
ILD	90	1462	5.8	2.19	Myelosuppression	15	534	2.73	4.94
				(1.77–2.71)					(2.96-8.26)
Malaise	15	1537	0.97	2.03	Neutrophil count decreased	25	524	4.55	3.21
				(1.22–3.38)					(2.15–4.80)
Pancytopenia	20	1532	1.29	2.02					
				(1.30–3.14)					
Death	14	1538	0.9	1.87					
				(1.10–3.17)					
Platelet count decreased	49	1503	3.16	1.83					
				(1.38–2.43)					
Vomiting	15	1537	0.97	1.77					
				(1.06–2.95)					
Neutropenia	24	1528	1.55	1.38					
				(0.92–2.06)					
Hemoglobin decreased	11	1541	0.71	1.35					
				(0.75–2.44)					
Renal impairment	17	1535	1.1	1.02					
				(0.63–1.65)					
Decreased appetite	11	1541	0.71	0.93					
				(0.51–1.68)					
Pyrexia	17	1535	1.1	0.79					
				(0.49–1.27)					
Febrile neutropenia	12	1540	0.77	0.74					
				(0.42–1.31)					
White blood cell count decreased	14	1538	0.9	0.67					
				(0.40–1.14)					

Notes: ROR: reported odds ratio. 95% CI: 95% confidence interval. MDS: myelodysplastic syndrome. AML: acute myeloid leukemia. ILD: interstitial lung disease.

**Table 2:** Proportion of post-event outcomes for the 15 adverse events associated with olaparib.

Adverse Events	Case (n)	Post-Event Outcome					
		Recovered	Remission	Not recovered	With Sequelae	Death	Unclear
**(A) Hematological-related AEs**							
Anemia	496	223 (45.0%)	93 (18.8%)	46 (9.2%)	0 (0%)	1 (0.2%)	133 (26.8%)
MDS	30	0 (0%)	1 (3.3%)	14 (46.7%)	0 (0%)	8 (26.7%)	7 (23.3%)
AML	12	0 (0%)	0 (0%)	3 (25.0%)	0 (0%)	6 (50.0%)	3 (25.0%)
Myelosuppression	33	12 (36.3%)	2 (6.1%)	2 (6.1%)	0 (0%)	0 (0%)	17 (51.5%)
Neutrophil count decreased	63	23 (36.5%)	8 (12.7%)	3 (4.8%)	0 (0%)	0 (0%)	29 (46.0%)
Platelet count decreased	49	26 (53.1%)	8 (16.3%)	3 (6.1%)	0 (0%)	2 (4.1 %)	10 (20.4%)
Pancytopenia	20	8 (40.0%)	4 (20.0%)	1 (5.0%)	0 (0%)	0 (0%)	7 (35.0%)
**(B) Gastrointestinal disorders**							
Ileus	14	2 (14.3%)	1 (7.1%)	1 (7.1%)	0 (0%)	0 (0%)	10 (71.5%)
Nausea	34	14 (41.2%)	6 (17.6%)	7 (20.6%)	0 (0%)	0 (0%)	7 (20.6%)
Vomiting	15	6 (40.0%)	4 (26.7%)	2 (13.3%)	0 (0%)	0 (0%)	3 (20.0%)
**(C) Others**							
Malignant neoplasm progression	242	0 (0%)	0 (0%)	1 (0.4%)	0 (0%)	32 (13.2%)	209 (86.4%)
Fatigue	5	4 (80.0%)	0 (0%)	1 (20.0%)	0 (0%)	0 (0%)	0 (0%)
ILD	90	37 (41.1%)	31 (34.5%)	2 (2.2%)	2 (2.2%)	0 (0%)	18 (20.0%)
Malaise	15	7 (46.7%)	3 (20.0%)	2 (13.3%)	0 (0%)	0 (0%)	3 (20.0%)
Death	14	0 (0%)	0 (0%)	0 (0%)	0 (0%)	14 (100.0%)	0 (0%)

Notes: For items for which an AE signal was detected, the analysis was performed using data for which information on outcomes after the onset of the AE was reported. MDS: myelodysplastic syndrome. AML: acute myeloid leukemia. ILD: interstitial lung disease.

**Table 3:** Proportion of post-event outcomes for the 11 AEs associated with niraparib.

Adverse Events	Case (n)	Post-Event Outcome					
		Recovered	Remission	Not Recovered	With Sequelae	Death	Unclear
**(A) Hematological-related AEs**							
Thrombocytopenia	44	20 (45.4%)	7 (15.9%)	8 (18.2%)	0 (0%)	0 (0%)	9 (20.5%)
Platelet count decreased	75	36 (48.0%)	13 (17.3%)	17 (22.7%)	0 (0%)	0 (0%)	9 (12.0%)
Anemia	39	14 (35.9%)	10 (25.6%)	8 (20.5%)	0 (0%)	0 (0%)	7 (18.0%)
Myelosuppression	15	2 (13.3%)	1 (6.7%)	2 (13.3%)	0 (0%)	0 (0%)	10 (66.7%)
Neutrophil count decreased	25	12 (48.0%)	5 (20.0%)	6 (24.0%)	0 (0%)	0 (0%)	2 (8.0%)
**(B) Gastrointestinal disorders**							
Ileus	11	3 (27.3%)	1 (9.1%)	4 (36.3%)	0 (0%)	0 (0%)	3 (27.3%)
**(C) Others**							
Ovarian cancer recurrent	26	1 (3.8%)	1 (3.8%)	7 (27.0%)	0 (0%)	2 (7.7%)	15 (57.7%)
Ovarian cancer	35	1 (2.8%)	1 (2.8%)	15 (42.9%)	0 (0%)	3 (8.6%)	15 (42.9%)
Disease progression	57	2 (3.5%)	3 (3.5%)	21 (36.8%)	0 (0%)	4 (7.0%)	28 (49.2%)
Condition aggravated	10	0 (0%)	0 (0%)	4 (40.0%)	0 (0%)	2 (20.0%)	4 (40.0%)
Renal impairment	41	8 (19.5%)	3 (7.3%)	13 (31.7%)	0 (0%)	0 (0.0%)	17 (41.5%)

Notes: For the AEs for which a signal was detected in the previous analysis, the analysis was performed using data for which information on the outcome after the onset of the AE had been reported.

**Table 4:** Weibull parameter of 15 AEs with olaparib.

Adverse Events	Case (n)	Scale Parameter	Shape Parameter
		α (95% CI)	β (95% CI)
**(A) Hematological-related AEs**			
Anemia	327	84.54 (76.42–93.39)	1.15 (1.06–1.24)
MDS	18	361.28 (241.26–527.09)	1.33 (0.88–1.88)
AML	4	363.24 (181.38–708.13)	2.06 (0.77–4.21)
Myelosuppression	15	74.67 (40.29–134.06)	0.96 (0.60–1.40)
Neutrophil count decreased	31	55.16 (33.81–88.03)	0.80 (0.60–1.03)
Platelet count decreased	41	60.93 (40.87–89.39)	0.85 (0.67–1.04)
Pancytopenia	14	118.11 (68.90–196.54)	1.13 (0.74–1.59)
**(B) Gastrointestinal disorders**			
Ileus	5	65.97 (21.34–193.61)	1.08 (0.45–2.09)
Nausea	27	9.60 (4.39–20.21)	0.54 (0.41–0.70)
Vomiting	14	6.68 (2.04–20.49)	0.51 (0.34–0.72)
**(C) Others**			
Malignant neoplasm progression	24	187.71 (134.30–257.64)	1.35 (0.97–1.81)
Fatigue	8	34.74 (14.52–79.09)	0.98 (0.53–1.57)
ILD	67	151.25 (126.37–179.90)	1.46 (1.21–1.73)
Malaise	13	35.78 (13.60–88.19)	0.67 (0.41–1.01)
Death	2	397.34 (210.89–772.73)	4.17 (0.91–11.26)

Notes: 95% CI: 95% confidence interval. AEs: adverse events.

**Table 5:** Weibull parameter of selected 11 AEs with niraparib.

Adverse Events	Case (n)	Scale Parameter	Shape Parameter
		α (95% CI)	β (95% CI)
**(A) Hematological-related AEs**			
Thrombocytopenia	34	46.55 (32.96–64.74)	1.08 (0.82–1.37)
Platelet count decreased	65	53.20 (43.52–64.62)	1.31 (1.07–1.57)
Anemia	30	49.45 (35.05–68.73)	1.16 (0.86–1.50)
Myelosuppression	8	57.31 (30.39–103.86)	1.34 (0.74–2.11)
Neutrophil count decreased	23	47.37 (32.47–66.99)	1.24 (0.87–1.67)
**(B) Gastrointestinal disorders**			
Ileus	10	53.39 (36.08–77.07)	1.90 (1.12–2.88)
**(C) Others**			
Ovarian cancer recurrent	20	41.94 (30.03–57.51)	1.50 (1.05–2.01)
Ovarian cancer	28	67.74 (47.49–95.05)	1.17 (0.85–1.53)
Disease progression	43	55.77 (42.50–72.42)	1.21 (0.95–1.49)
Condition aggravated	8	50.81 (29.50–84.69)	1.56 (0.87–2.44)
Renal impairment	32	46.34 (31.42–67.17)	0.99 (0.75–1.27)

Notes: 95% CI: 95% confidence interval. AEs: adverse events.

## Data Availability

The data that support the findings of this study are available at https://www.info.pmda.go.jp/fukusayoudb/CsvDownload.jsp (accessed on 6 June 2022).

## References

[1] (2022). Cancer Statistics in Japan. https://ganjoho.jp/public/qa_links/report/statistics/2022_en.html.

[2] da Cunha Colombo Bonadio R.R., Fogace R.N., Miranda V.C., Diz M.D.P.E. (2018). Homologous recombination deficiency in ovarian cancer: A review of its epidemiology and management. Clinics.

[3] Enomoto T., Aoki D., Hattori K., Jinushi M., Kigawa J., Takeshima N., Tsuda H., Watanabe Y., Yoshihara K., Sugiyama T. (2019). The first Japanese nationwide multicenter study of BRCA mutation testing in ovarian cancer: CHARacterizing the cross-sectionaL approach to Ovarian cancer geneTic TEsting of BRCA (CHARLOTTE). Int. J. Gynecol. Cancer.

[4] Hirasawa A., Imoto I., Naruto T., Akahane T., Yamagami W., Nomura H., Masuda K., Susumu N., Tsuda H., Aoki D. (2017). Prevalence of pathogenic germline variants detected by multigene sequencing in unselected Japanese patients with ovarian cancer. Oncotarget.

[5] Norquist B.M., Harrell M.I., Brady M.F., Walsh T., Lee M.K., Gulsuner S., Bernards S.S., Casadei S., Yi Q., Burger R.A. (2016). Inherited Mutations in Women With Ovarian Carcinoma. JAMA Oncol..

[6] BRCA1- and BRCA2-Associated Hereditary Breast and Ovarian Cancer. https://www.ncbi.nlm.nih.gov/books/NBK1247/.

[7] Erkko H., Xia B., Nikkilä J., Schleutker J., Syrjäkoski K., Mannermaa A., Kallioniemi A., Pylkäs K., Karppinen S.M., Rapakko K. (2007). A recurrent mutation in PALB2 in Finnish cancer families. Nature.

[8] Renwick A., Thompson D., Seal S., Kelly P., Chagtai T., Ahmed M., North B., Jayatilake H., Barfoot R., Spanova K. (2006). ATM mutations that cause ataxia-telangiectasia are breast cancer susceptibility alleles. Nat. Genet..

[9] Weischer M., Bojesen S.E., Ellervik C., Tybjaerg-Hansen A., Nordestgaard B.G. (2008). CHEK2*1100delC genotyping for clinical assessment of breast cancer risk: Meta-analyses of 26,000 patient cases and 27,000 controls. J. Clin. Oncol..

[10] Seal S., Thompson D., Renwick A., Elliott A., Kelly P., Barfoot R., Chagtai T., Jayatilake H., Ahmed M., Spanova K. (2006). Truncating mutations in the Fanconi anemia J gene BRIP1 are low-penetrance breast cancer susceptibility alleles. Nat. Genet..

[11] Meindl A., Hellebrand H., Wiek C., Erven V., Wappenschmidt B., Niederacher D., Freund M., Lichtner P., Hartmann L., Schaal H. (2010). Germline mutations in breast and ovarian cancer pedigrees establish RAD51C as a human cancer susceptibility gene. Nat. Genet..

[12] Tew W.P., Lacchetti C., Ellis A., Maxian K., Banerjee S., Bookman M., Jones M.B., Lee J.M., Lheureux S., Liu J.F. (2020). PARP Inhibitors in the Management of Ovarian Cancer: ASCO Guideline. J. Clin. Oncol..

[13] Sunada S., Nakanishi A., Miki Y. (2018). Crosstalk of DNA double-strand break repair pathways in poly(ADP-ribose) polymerase inhibitor treatment of breast cancer susceptibility gene 1/2-mutated cancer. Cancer Sci..

[14] Moore K., Colombo N., Scambia G., Kim B.G., Oaknin A., Friedlander M., Lisyanskaya A., Floquet A., Leary A., Sonke G.S. (2018). Maintenance Olaparib in Patients with Newly Diagnosed Advanced Ovarian Cancer. N. Engl. J. Med..

[15] Pujade-Lauraine E., Ledermann J.A., Selle F., Gebski V., Penson R.T., Oza A.M., Korach J., Huzarski T., Poveda A., Pignata S. (2017). Olaparib tablets as maintenance therapy in patients with platinum-sensitive, relapsed ovarian cancer and a BRCA1/2 mutation (SOLO2/ENGOT-Ov21): A double-blind, randomised, placebo-controlled, phase 3 trial. Lancet Oncol..

[16] Ray-Coquard I., Pautier P., Pignata S., Pérol D., González-Martín A., Berger R., Fujiwara K., Vergote I., Colombo N., Mäenpää J. (2019). Olaparib plus Bevacizumab as First-Line Maintenance in Ovarian Cancer. N. Engl. J. Med..

[17] Robson M., Im S.A., Senkus E., Xu B., Domchek S.M., Masuda N., Delaloge S., Li W., Tung N., Armstrong A. (2017). Olaparib for Metastatic Breast Cancer in Patients with a Germline BRCA Mutation. N. Engl. J. Med..

[18] González-Martín A., Pothuri B., Vergote I., DePont Christensen R., Graybill W., Mirza M.R., McCormick C., Lorusso D., Hoskins P., Freyer G. (2019). Niraparib in Patients with Newly Diagnosed Advanced Ovarian Cancer. N. Engl. J. Med..

[19] Mirza M.R., Monk B.J., Herrstedt J., Oza A.M., Mahner S., Redondo A., Fabbro M., Ledermann J.A., Lorusso D., Vergote I. (2016). Niraparib Maintenance Therapy in Platinum-Sensitive, Recurrent Ovarian Cancer. N. Engl. J. Med..

[20] de Bono J., Mateo J., Fizazi K., Saad F., Shore N., Sandhu S., Chi K.N., Sartor O., Agarwal N., Olmos D. (2020). Olaparib for Metastatic Castration-Resistant Prostate Cancer. N. Engl. J. Med..

[21] Golan T., Hammel P., Reni M., Van Cutsem E., Macarulla T., Hall M.J., Park J.O., Hochhauser D., Arnold D., Oh D.Y. (2019). Maintenance Olaparib for Germline. N. Engl. J. Med..

[22] Moore K.N., Secord A.A., Geller M.A., Miller D.S., Cloven N., Fleming G.F., Wahner Hendrickson A.E., Azodi M., DiSilvestro P., Oza A.M. (2019). Niraparib monotherapy for late-line treatment of ovarian cancer (QUADRA): A multicentre, open-label, single-arm, phase 2 trial. Lancet Oncol..

[23] Gallagher J.R., Heap K.J., Carroll S., Travers K., Harrow B., Westin S.N. (2019). Real-world adverse events with niraparib 200 mg/day maintenance therapy in ovarian cancer: A retrospective study. Future Oncol..

[24] Matulonis U.A., Walder L., Nøttrup T.J., Bessette P., Mahner S., Gil-Martin M., Kalbacher E., Ledermann J.A., Wenham R.M., Woie K. (2019). Niraparib Maintenance Treatment Improves Time Without Symptoms or Toxicity (TWiST) Versus Routine Surveillance in Recurrent Ovarian Cancer: A TWiST Analysis of the ENGOT-OV16/NOVA Trial. J. Clin. Oncol..

[25] LaFargue C.J., Dal Molin G.Z., Sood A.K., Coleman R.L. (2019). Exploring and comparing adverse events between PARP inhibitors. Lancet Oncol..

[26] Tian X., Chen L., Gai D., He S., Jiang X., Zhang N. (2022). Adverse Event Profiles of PARP Inhibitors: Analysis of Spontaneous Reports Submitted to FAERS. Front. Pharmacol..

[27] Gao Q., Zhu J., Zhao W., Huang Y., An R., Zheng H., Qu P., Wang L., Zhou Q., Wang D. (2022). Olaparib Maintenance Monotherapy in Asian Patients with Platinum-Sensitive Relapsed Ovarian Cancer: Phase III Trial (L-MOCA). Clin. Cancer Res..

[28] van Puijenbroek E.P., Egberts A.C., Heerdink E.R., Leufkens H.G. (2000). Detecting drug-drug interactions using a database for spontaneous adverse drug reactions: An example with diuretics and non-steroidal anti-inflammatory drugs. Eur. J. Clin. Pharmacol..

[29] Sauzet O., Carvajal A., Escudero A., Molokhia M., Cornelius V.R. (2013). Illustration of the weibull shape parameter signal detection tool using electronic healthcare record data. Drug. Saf..

[30] Wang C., Li J. (2021). Haematologic toxicities with PARP inhibitors in cancer patients: An up-to-date meta-analysis of 29 randomized controlled trials. J. Clin. Pharm. Ther..

[31] Morice P.M., Chrétien B., Da Silva A., Dolladille C., Alexandre J. (2021). Occurrence of Pancytopenia Among Patients With Cancer Treated With Poly(Adenosine Diphosphate-Ribose) Polymerase Inhibitors: A Pharmacoepidemiologic Study. JAMA Oncol..

[32] Shu Y., Ding Y., He X., Liu Y., Wu P., Zhang Q. Hematological toxicities in PARP inhibitors: A real-world study using FDA adverse event reporting system (FAERS) database. Cancer Med..

[33] Stemmer A., Shafran I., Stemmer S.M., Tsoref D. (2020). Comparison of Poly (ADP-ribose) Polymerase Inhibitors (PARPis) as Maintenance Therapy for Platinum-Sensitive Ovarian Cancer: Systematic Review and Network Meta-Analysis. Cancers.

[34] Berek J.S., Matulonis U.A., Peen U., Ghatage P., Mahner S., Redondo A., Lesoin A., Colombo N., Vergote I., Rosengarten O. (2018). Safety and dose modification for patients receiving niraparib. Ann. Oncol..

[35] Gonzalez A., Mirza M., Vergote I., Li Y., Hazard S., Clark R., Graybill W., Pothuri B., Monk B. (2018). A prospective evaluation of tolerability of niraparib dosing based upon baseline body weight (wt) and platelet (blplt) count: Blinded pooled interim safety data from the PRIMA Study. Ann. Oncol..

[36] Morice P.M., Leary A., Dolladille C., Chrétien B., Poulain L., González-Martín A., Moore K., O’Reilly E.M., Ray-Coquard I., Alexandre J. (2021). Myelodysplastic syndrome and acute myeloid leukaemia in patients treated with PARP inhibitors: A safety meta-analysis of randomised controlled trials and a retrospective study of the WHO pharmacovigilance database. Lancet Haematol..

[37] Hao J., Liu Y., Zhang T., He J., Zhao H., An R., Xue Y. (2021). Efficacy and safety of PARP inhibitors in the treatment of advanced ovarian cancer: An updated systematic review and meta-analysis of randomized controlled trials. Crit. Rev. Oncol. Hematol..

[38] Fujiwara K., Fujiwara H., Yoshida H., Satoh T., Yonemori K., Nagao S., Matsumoto T., Kobayashi H., Bourgeois H., Harter P. (2021). Olaparib plus bevacizumab as maintenance therapy in patients with newly diagnosed, advanced ovarian cancer: Japan subset from the PAOLA-1/ENGOT-ov25 trial. J. Gynecol. Oncol..

[39] Shenolikar R., Durden E., Meyer N., Lenhart G., Moore K. (2018). Incidence of secondary myelodysplastic syndrome (MDS) and acute myeloid leukemia (AML) in patients with ovarian or breast cancer in a real-world setting in the United States. Gynecol. Oncol..

[40] Morton L.M., Dores G.M., Schonfeld S.J., Linet M.S., Sigel B.S., Lam C.J.K., Tucker M.A., Curtis R.E. (2019). Association of Chemotherapy for Solid Tumors With Development of Therapy-Related Myelodysplastic Syndrome or Acute Myeloid Leukemia in the Modern Era. JAMA Oncol..

[41] NCCN Clinical Practice Guidelines in Oncology (NCCN Guidelines®) Antiemesis. https://www.nccn.org/professionals/physician_gls/pdf/antiemesis.pdf.

[42] Sun W., Li J., Zhang Z., Su X. (2021). Gastrointestinal events with PARP inhibitors in cancer patients: A meta-analysis of phase II/III randomized controlled trials. J. Clin. Pharm. Ther..

[43] Moore K.N., Mirza M.R., Matulonis U.A. (2018). The poly (ADP ribose) polymerase inhibitor niraparib: Management of toxicities. Gynecol. Oncol..

[44] PLoSker G.L., Goa K.L. (1991). Granisetron. A review of its pharmacological properties and therapeutic use as an antiemetic. Drugs.

[45] Liu Y., Meng J., Wang G. (2018). Risk of selected gastrointestinal toxicities associated with poly (ADP-ribose) polymerase (PARP) inhibitors in the treatment of ovarian cancer: A meta-analysis of published trials. Drug. Des. Devel. Ther..

[46] Satake R., Matsumoto K., Tanaka M., Mukai R., Shimada K., Yoshida Y., Inoue M., Hasegawa S., Iguchi K., Ikesue H. (2021). Analysis of Drug-Induced Gastrointestinal Obstruction and Perforation Using the Japanese Adverse Drug Event Report Database. Front. Pharmacol..

[47] Ledermann J., Harter P., Gourley C., Friedlander M., Vergote I., Rustin G., Scott C., Meier W., Shapira-Frommer R., Safra T. (2012). Olaparib maintenance therapy in platinum-sensitive relapsed ovarian cancer. N. Engl. J. Med..

[48] Valabrega G., Pothuri B., Oaknin A., Graybill W., Sánchez A.B., Mccormick C., Baurain J., Hoskins P., Denys H., O’Cearbhaill R.E. (2020). Efficacy and safety of niraparib in older patients (pts) with advanced ovarian cancer (OC): Results from the PRIMA/ENGOT-OV26/GOG-3012 trial. Ann. Oncol..

[49] Ma Z., Sun X., Zhao Z., Lu W., Guo Q., Wang S., You J., Zhang Y., Liu L. (2021). Risk of pneumonitis in cancer patients treated with PARP inhibitors: A meta-analysis of randomized controlled trials and a pharmacovigilance study of the FAERS database. Gynecol. Oncol..

